# Assessing the efficacy and safety of Juan Bi Tang for dialysis-related myofascial pain in the fistula arm: Study protocol for a randomized cross-over trial

**DOI:** 10.3389/fpubh.2022.925232

**Published:** 2022-08-19

**Authors:** Yung-Tang Hsu, Hwee-Yeong Ng, Yung-Hsiang Chen, Yu-Chuen Huang, Yan-Yuh Lee, Ming-Yen Tsai

**Affiliations:** ^1^Department of Chinese Medicine, Kaohsiung Chang Gung Memorial Hospital and Chang Gung University College of Medicine, Kaohsiung, Taiwan; ^2^Division of Nephrology, Department of Internal Medicine, Kaohsiung Medical University, Kaohsiung, Taiwan; ^3^Graduate Institute of Integrated Medicine, College of Chinese Medicine, Research Center for Chinese Medicine and Acupuncture, China Medical University, Taichung, Taiwan; ^4^Department of Psychology, College of Medical and Health Science, Asia University, Taichung, Taiwan; ^5^Department of Medical Research, China Medical University Hospital and School of Chinese Medicine, China Medical University, Taichung, Taiwan; ^6^Department of Physical Medicine and Rehabilitation, Kaohsiung Chang Gung Memorial Hospital and Chang Gung University College of Medicine, Kaohsiung, Taiwan

**Keywords:** hemodialysis, Chinese herbal medicine, randomized trial, myofascial pain, study protocol

## Abstract

**Background:**

Dialysis-related myofascial pain in hemodialysis (HD) patients is an important issue that is associated with many other psychosomatic problems. Effective interventions are required to alleviate pain in this group. Chinese herbal medicine (CHM) may be a potential therapeutic treatment for reducing pain. The aim of this study is to evaluate the effects of a classic CHM formula intervention on pain intensity, daily function, quality of life (QOL), and safety in patients receiving HD in a dialysis center within the context of southern Taiwan.

**Methods:**

This will be a randomized, open label, cross-over trial with two parallel groups in a pre- and post-test study. Forty patients reporting myofascial pain related to the arteriovenous (AV) fistula in the arm during regular HD sessions will be recruited. Participants will receive 4 weeks of treatment with Juan Bi Tang (JBT) and 4 weeks of no treatment in a random order, separated by a washout period of 2 weeks. Treatment doses (3 g JBT) will be consumed thrice daily. The primary outcome measure will be the Kidney Disease Quality of Life 36-Item Short-Form Survey. Secondary outcomes will include the Fugl-Meyer Assessment-arm, Visual Analogue Scale (VAS) of pain, and grip strength. Outcomes will be collected before and after each intervention, for a total of four times per participant. The safety evaluation will focus on adverse events (AEs).

**Discussion:**

This study will be the first to use CHM to treat patients receiving HD with dialysis-related myofascial pain in their fistula arm and to perform a complete assessment of the treatment, including records of QOL, arm function and muscle power, severity of pain, and safety. The results of the study will provide convincing evidence on the use of JBT as an adjuvant treatment for dialysis-related myofascial pain.

**Trial registration:**

Clinicaltrials.gov registry (NCT04417101) registered 30 May 2020.

## Background

Myofascial pain syndrome (MPS) is characterized by localized pain (1), paresthesia, exquisite tenderness, restricted range of motion, and hypersensitivity at specific anatomic sites, which are termed taut bands with active myofascial trigger points (MTrPs) (2–4). According to the International Association for the Study of Pain and the American Academy of Pain Medicine, the essential criteria for the diagnosis of MPS are hypersensitive spots that cause local pain and symptoms that can be recreated by palpation (5). In the United States, about 9 million people have MPS (6), and its prevalence in the general population is between 9 and 85% (5, 7).

Myofascial pain syndrome is often more serious in females than in males, and its prevalence apparently increases with age (8). Currently, the etiology of MPS is poorly understood, but various aspects of its pathogenesis are being investigated (8, 9). The negative effects of MPS on quality of life (QOL), such as loss of work tolerance, fatigue, and weakness, are well-documented (10). However, few studies have focused on MPS in specific populations.

One major complication of chronic kidney disease (CKD) or end stage kidney disease (ESRD) is rheumatic disorders, and over half (60%) of patients receiving hemodialysis (HD) develop musculoskeletal disorders (11). Therefore, it is important to consider a differential diagnosis of MPS in these patients. Another form of MPS which sometimes develops in patients receiving HD is related to the fistula arm. In such cases, the symptoms are induced by factors implicated in or related to maintenance HD.

Factors such as comorbidities, dialysis type, metabolic disorders, nutritional factors, biomechanical imbalance, and/or physio-psychological deconditioning may simultaneously contribute to the development of dialysis-related myofascial pain (12, 13). However, the pathological mechanisms involved are not well-known. Currently, the underlying pathologies are considered to be local injury from gross venipuncture or constant micro-trauma from venous pressure during each HD session in the affected arm muscle (8, 14). This stress can lead to inappropriate acetylcholine (Ach) activity at the endplate. Such activity can cause an energy crisis that favors the release of nociceptive neurotransmitters. The altered ACh in turn triggers an active contractile phenomenon (taut bands), and the nociceptive neurotransmitters, which compensate for tissue hypoxemia, cause pain neurotransmission or sensations in the form of local pain and referred pain (15–17).

There is no definitive treatment for dialysis-related myofascial pain, and the most common treatment modality is based on condition-specific and stepwise pain management for ESRD (18, 19). Aspects of dialysis-related myofascial pain can be easily mitigated with non-steroidal anti-inflammatory drugs (NSAIDs), Cyclooxygenase-2 (COX-2) inhibitors or topical analgesics (18). One problem with this approach to pain management is that the use of oral analgesics may have side effects such as bleeding, fluid retention, and cardiovascular events; thus, they are not appropriate for all patients (20). In addition, some sedative-hypnotic agents or muscle relaxants for alleviation of symptoms of anxiety and/or depression carry risks of hypotension and physical dependence (21, 22).

The aforementioned limitations of conventional treatment have led to the present study, which advocates the use of Chinese herbal medicine (CHM) therapy for pain management. CHM therapy is safe and inexpensive, and it can alleviate the pain and anxiety associated with several diseases (23, 24). Moreover, CHM use by patients with CKD has positive results on reducing the risk of ESRD, as determined by thorough analysis of the classification of CHM prescriptions in the Taiwan National Health Insurance Research Database (25). Juan Bi Tang (JBT) has a long history of use as a classic herbal prescription for treating Bi syndrome and was first documented in a classical Chinese medical book, *Prescriptions for Ji Sheng Fang* (CE 1253). As joint pain is one of the most common symptoms of Bi syndrome, some scholars often translate it as “Arthralgia syndrome.” Clinically, Bi syndrome covers many musculoskeletal diseases, such as rheumatoid arthritis, osteoarthritis, rheumatism, fibromyalgia, or myofascial syndrome (26). The basic pathology of Bi syndrome is the obstruction of *qi* (also called vital energy) and blood in the meridians due to the invasion of pathogenic wind, cold, and dampness. According to traditional Chinese medicine (TCM), open meridians and normal circulation of the *qi* and blood are associated with an absence of pain, while obstruction of the meridians and the flow of *qi* and blood are associated with the presence of pain. Juan Bi Tang is used for treating pain in the upper limbs by warming up meridians so as to dissipate cold, eliminate dampness, activate the *qi* and blood, and also resolve stasis (27). Although JBT is widely used in the treatment of musculoskeletal disorders, no studies have been conducted to examine its efficacy and safety for the treatment of dialysis-related myofascial pain in patients receiving HD. This randomized, open label, parallel-group, cross-over, single center clinical trial will aim to determine the possible benefits of JBT in patients with dialysis-related myofascial pain.

## Methods/Design

### Study design

**Figure 1:**
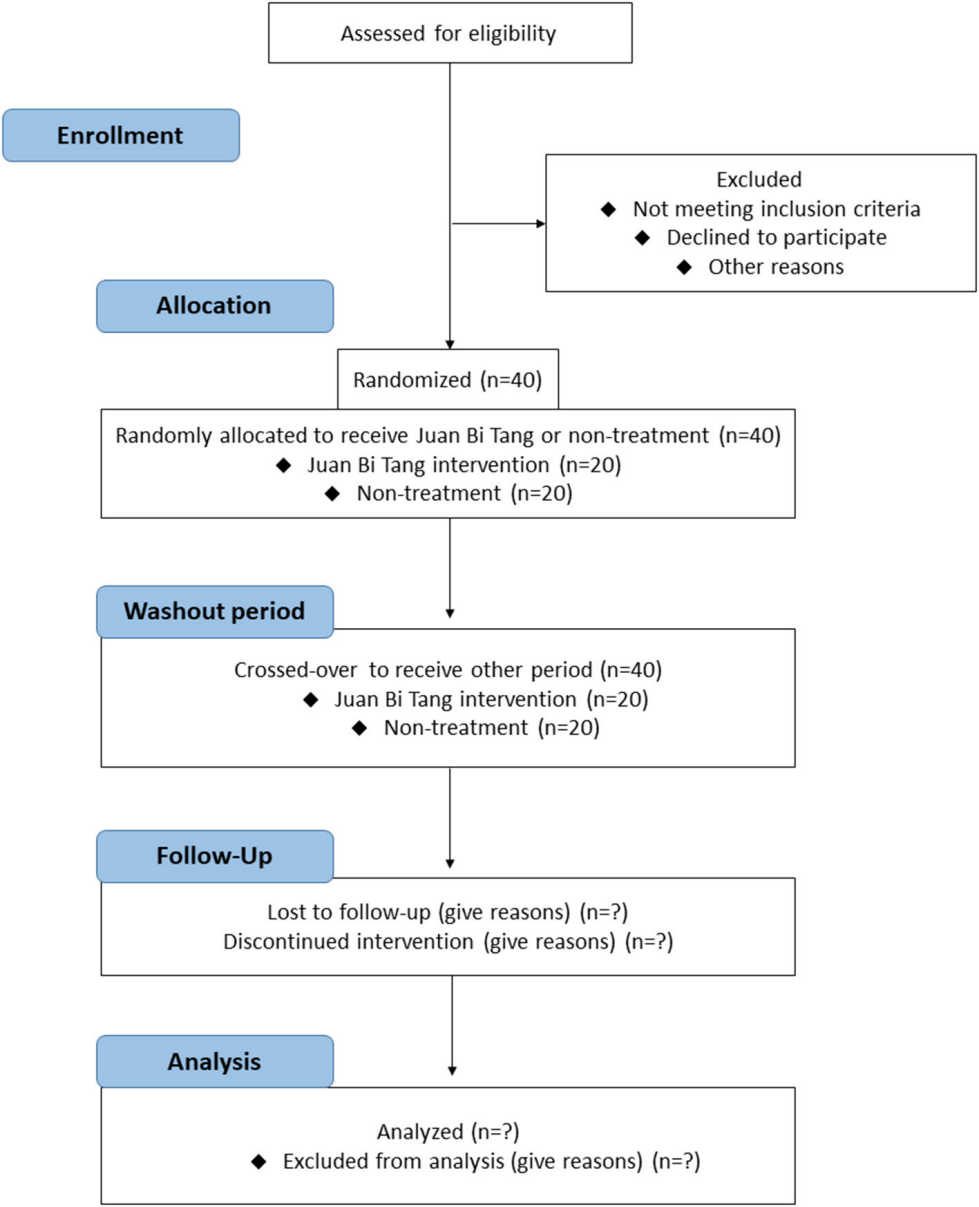
Study design from enrollment to analysis following the CONSORT 2010 flow diagram.

This single-center, two-phase study will last 12 weeks. It will be a randomized, prospective, crossover, open-label study comprising two groups and two treatment sequences, each consisting of 4 weeks of daily treatments separated by a 2-week washout period (Figure 1). In total, 40 patients will be recruited (20 patients per group) from a dialysis center in Chang Gung Memorial Hospital (CGMH), Kaohsiung, Taiwan. The subjects will be randomly and equally assigned with a central registration method into group A (JBT course in period I but not in period II) or group B (JBT course not in period I but in period II). The research protocol has been approved by the Ethics Committee of the CGMH (202000477A3) and registered at ClinicalTrials.gov (NCT04417101). The Standard Protocol Items: Recommendation for Interventional Trials (SPIRIT) 2013 checklist is provided in Table 1.

**Table 1:** Summary of collected data at each time point according to SPIRIT 2013 guidelines.

**Testing variables**	**Screening**	**Randomization**				**Follow-up**
	**Week 1**	**Week 0 (baseline)**	**Week 4**	**Week 6**	**Week 10**	**Week 12**
Inclusion and exclusion criteria	X					
Baseline information	X					
Inform consent	X					
Intervention						
Period I		X	X			
Period II				X	X	
Assessment						
VAS		X	X	X	X	X
KDQOL-36		X	X	X	X	X
FMA-UE		X	X	X	X	X
Griping force		X	X	X	X	X
Laboratory data		X	X	X	X	X
Conditional information			X	X	X	
Adverse events			X	X	X	X

VAS, visual analog scale; KDQOL-36, Kidney Disease Quality of Life 36-Item Short-Form Survey; FMA-UE, Fugl-Meyer Assessment for upper extremity.

### Study participants

Participants will be recruited from Kaohsiung CGMH via posters and advertisements on the official hospital website. The recruitment period is planned to last 18 months. All participants will provide informed consent before randomization. The schedule of patient enrollment, intervention, and assessment is illustrated in Table 1.

### Eligibility criteria

#### Inclusion criteria

**Figure 2:**
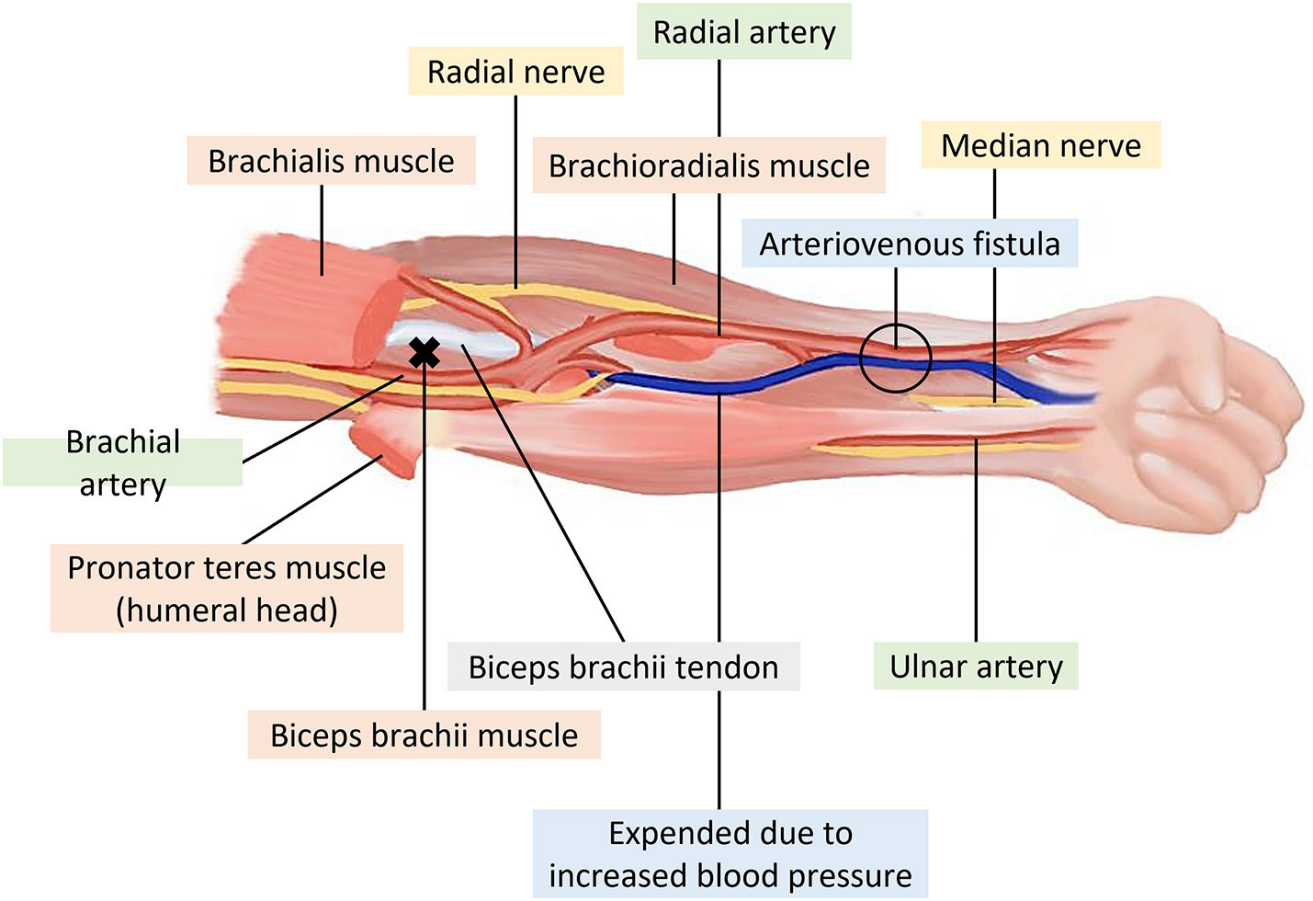
Muscles of the anterior forearm around the arteriovenous fistula (x: trigger points of the biceps brachii).

Participants meeting the following criteria are eligible:

1. Age of 20 years or older;
2. Treatment with conventional HD three times a week via an arteriovenous (AV) fistula for at least 3 months;
3. Myofascial trigger points in one or more muscles around the AV fistula, namely, the brachioradialis, the flexor carpi radialis, the palmaris longus, and/or the pronator teres (Figure 2), accompanied by dialysis-related myofascial pain presenting during HD and diagnosed by a nephrologist (28);
4. Onset of symptoms within 1 month before enrollment;
5. Exquisite tenderness on palpation in the taut band, with moderate intensity of pain at baseline (i.e., a pain score >3 on a numeric rating scale);
6. No change of painkillers, muscle relaxants, or anti-inflammatory medications and no use of topical anesthetics in the past week.

#### Exclusion criteria

The exclusion criteria are as follows:

1. Severe systemic disease (i.e., sepsis, cancer, coagulopathy) interfering with therapy attendance;
2. Comorbid conditions such as rheumatoid arthritis, stroke, chronic liver disease, radiculopathies of the upper limb, recent history of cervical/shoulder/arm surgery, or trauma;
3. Depression and/or presence of a psychiatric disorder;
4. Treatment with CHM for dialysis-related myofascial pain in the past month;
5. Allergic reactions (i.e., skin rash, purpura, and vasculitis) to CHM;
6. Inability to comprehend or sign an informed consent form.

#### Subject withdrawal criteria

Study participation will be terminated under the following conditions:

1. Occurrence of severe complications and/or general health deterioration (i.e., sepsis, bleeding, AV fistula occlusion, and progress of electrolyte disorders such as ECG change of hyperkalemia, or uncontrolled calcium × phosphorus (Ca × P) level);
2. Occurrence of a severe adverse event (AE) of any type during the intervention;
3. Voluntary withdrawal from the trial, absence from two consecutive interviews, or loss to follow-up.

### Interventions

The CHM product investigated in this study will be prescription-grade JBT powder (Product code; NO.0321131, Ko Da Pharmaceutical Co., Ltd., Taoyuan, Taiwan) manufactured in accordance with Good Manufacturing Practices (GMP) and tested before factory release. Juan Bi Tang consists of 4 g *Rx. Angelica Sinensis*當歸, 4 g *Rx. Paeoniae Rubra*赤芍, 4 g *Rx. Astragali*黃耆, 4 g *Rz. Curcuma Longae*薑黃, 1 g *Rx. et Rz. Notopterygii*羌活, 4 g *Rx. Saposhnikoviae*防風, 3 g *Rz. Zingiberis Recens*生薑, 2 g *Fr. Zizyphi Jujube*大棗, and 1.5 g *Rx. Glycyrrhizae Preparata*炙甘草. These raw herb materials, together with starch excipient, are extracted and concentrated according to a standardized Chinese formula (see: http://www.koda.com.tw/tqm04_e.aspx). The participants will be instructed to take JBT orally in doses of 3 g (per bag) each time, thrice daily, for 4 weeks. Patients will return each week to the outpatient clinic for evaluation, medication, and counting of any unconsumed bags. Weekly compliance below 75% will be considered a deviation from the protocol and will lead to exclusion from further participation in the study. Participants in the non-treatment period will receive simple self-care measures for MPS based on the non-pharmacologic intervention in patients with CKD by Pham et al. (18). These will mainly include physical activities such as stretching, aerobic and strength exercises, and some types of combined exercises (18). The duration of the intervention will be 8 weeks, and the follow-up will be at 2 weeks.

### Concomitant treatments

Routine medication for maintenance HD will be continued. The medication regimen will be recorded at every visit, and participants will need to inform the study assistant of any changes to their medication, supplements, and even exercise. Additional acupuncture, herbal prescriptions, or other interventions by other TCM practitioners will not be permitted during the treatment period.

### Randomization and allocation

The participants will be allocated into group A (*n* = 20) or group B (*n* = 20) according to a randomized list generated in Microsoft Excel 2016. Participants will be coded with identification numbers to guarantee confidentiality during data analysis. The physicians will have no access to the sequence, and the investigators will distribute the drugs. All members of the research team will be explicitly instructed not to inform the participants of their allocations. The clinical outcomes assessor will be blinded to the allocations to decrease the risk of observer bias. Participants' files will be kept in a box in a locked cabinet only accessible by an assistant until the end of the study.

Participants will be coded with identification numbers to guarantee confidentiality during data analysis. Participants' files will be stored in a secure room in the TCM Education Research Office, Rehabilitation Building of Kaohsiung CGMH.

### Outcome measurement

The subjects will be assessed five times: prior to the study, at 4 weeks (at the end of period I), at 6 weeks (at the beginning of period II), at 10 weeks (at the end of period II), and at follow-up (2 weeks after the trial period ends). A research assistant will be trained to ensure the accuracy of outcome assessments and data collection.

#### Primary outcome

The primary outcome used in this study will be the Kidney Disease Quality of Life 36-Item Short-Form Survey (KDQOL-36), a self-report measure developed for health-related concerns of individuals who visit dialysis facilities for treatment (29). The KDQOL-36 is available in English and was translated into Mandarin Chinese by the RAND Corporation (see: http://www.rand.org/health/surveys_tools/kdqol.html). The instrument is composed of 12 general health items and 24 kidney-specific items. The items on general health are summarized into a Physical Component Summary (PCS) score and a Mental Component Summary (MCS) score. The 24 kidney-specific items comprise three scales: symptoms and problems, burden of kidney disease, and effects of kidney disease (12, 4, and 8 items, respectively). The raw scores are transformed into a linear range of 0–100, where higher scores indicate better QOL (30).

#### Secondary outcomes

Secondary outcome measures will include arm motor function evaluated with the Fugl-Meyer Assessment for upper extremity (FMA-UE); muscle power threshold, measured with a grip algometer; and self-reported degree of pain [Visual Analogue Scale (VAS) score].

Upper limb function will be assessed with the FMA-UE, a stroke-specific test for measuring motor impairment and recovery (31). The FMA-UE, which is widely used to evaluate upper limb motor function in patients with stroke, is often applied in studies of pain disorders in the upper extremities (32). The maximum score of the upper extremity scale is 66 points, divided among three components: shoulder–arm (36 points), wrist–hand (24 points), and coordination (6 points). The reliability and validity of the FMA-UE are excellent, and it is sufficiently sensitive for both clinical and research applications (33). The assessment will be performed in a standardized manner to differentiate the function levels of the fistula and non-fistula arms.

Handgrip strength will be measured with a digital handgrip dynamometer (TTM-YD, Tokyo, Japan). Two consecutive measurements of the fistula-side hand will be performed at an interval of 30 s, and the maximal isometric reading will be used for data analysis (34). Hand strength is used as a simple metric of general muscle strength for identifying functional deficits (35). It can also provide good resolution for localized pathologies and provides outcomes that allow better understanding of the therapeutic response of the impaired hand (36).

Pain perception will be recorded immediately after cannulation on a VAS, a non-graduated horizontal line of 100 mm where 0 = no pain at all and 100 = as painful as possible. The VAS and grip strength measures will be assessed immediately before the first treatment; at 4, 6, and 10 weeks after the first treatment; and at follow-up.

### Safety assessments

Juan Bi Tang is described in the ancient literature of TCM as relatively safe, and previous clinical studies have reported no major toxicity in therapeutic doses (37–39). However, we will still perform a series of measures to assess the safety of JBT throughout the entire trial due to possible herb-induced toxicity or herb-dialysis interaction (40). All safety-related variables, including vital signs, physical examination, hematological test, biochemical test, and AEs, will be recorded in the case report at every visit. Laboratory testing of the participants' blood, electrolytes (i.e., potassium, phosphorus, and calcium), albumin, parathyroid hormone, and kidney and liver function will be monitored before the intervention, at 2-week intervals during the intervention, and again at 2 weeks after trial completion. In addition, all details of AEs and other ailments will also be documented accurately at every study visit, including the occurrence time, severity, duration, effective measures, and transfer. Each AE associated with the intervention drugs will be classified as mild, moderate, or severe. If severe AEs such as hyperkalemia, thrombocytopenia, liver injury, bleeding, or cardiovascular events related to JBT occur during the study, the principal investigator will provide immediate diagnosis and treatment and report the AE to the Institutional Review Board within 24 h of the time of recognition. All AEs will be monitored and recorded by an independent researcher unassociated with the study until recovery of the participant.

### Statistical analysis

#### Sample size

A statistical power analysis by G^*^Power version 3.1.9.2 for the differences in the outcome measure between both groups was conducted to determine the investigated sample size to achieve adequate power (80%, α = 0.05). For repeated measures ANOVA analysis, to achieve 80% power and a 25% effect size (40), at least 34 participants will be needed. Considering a 15% loss to follow-up, we plan to recruit 40 participants (20 participants for each group) to compensate for possible dropouts.

#### Data analyses

Both intention-to-treat (ITT) and per-protocol (PP) analyses will be applied in the present trial. Continuous data will be presented as mean ± SD, and categorical data will be presented as frequencies and proportions. The Student's *t*-test for continuous variables and chi-squared test for categorical variables will be used to compare the characteristics and clinical data of patients with and without JBT treatment. Repeated measures ANOVA will be conducted to compare the subjects' conditions over time. The Mann–Whitney U test and generalized estimating equation will be applied to compare differences when the distribution of data does not meet the assumption of normality. All analyses will be performed in IBM SPSS Statistics 22 (IBM Co., Armonk, NY, USA). A *p*-value <0.05 will be considered statistically significant.

## Discussion

Repeated myofascial pain in the fistula arm during HD sessions is a distressing symptom reported by patients receiving HD. Due to a lack of reporting in the past, this type of pain related to the fistula arm is rarely treated sufficiently to meet the needs of these patients. Uncontrolled pain in the affected muscles can degrade QOL and cause non-adherence to the recommended dialysis regimen, which can in turn increase the mortality rate due to consequent cardiovascular and pulmonary events (41, 42). Juan Bi Tang therapy uses an ancient herbal medicine to address these painful experiences, especially the inadequate flow of *qi* and blood. In a previous study, compared to Western medicine, JBT was found to be safe and particularly beneficial to arthritis patients with the cold and deficiency pattern (37). Most patients who receive HD have low physical performance and low immunity (43), and researchers have found that a feature of the TCM pattern in patients receiving HD is mainly deficiency syndrome (44). The etiology of TCM pays attention to the circulation of *qi* and blood, and insufficient or blocked circulation will cause pain. Juan Bi Tang is used clinically to treat painful obstruction of *qi* and blood deficiency. Thus, we propose the first pilot crossover trial to examine the effectiveness of JBT therapy in alleviating dialysis-related myofascial pain around the fistula arm in patients receiving HD.

At present, the findings of most animal studies have confirmed the benefits of JBT in improving arthritis and synovitis (45–47). Significant reductions in the serum levels of MMP-2 and MMP-9 indicate that JBT has a role to play in preventing osteoarthritis (45). Similar effects of JBT on synovial inflammation and bone destruction through inhibiting the pro-inflammatory cytokines have recently been reported by Wang et al. (46). In addition, a study by Zhao et al. has demonstrated that JBT ameliorates bone destruction and reduces bone loss induced by rheumatoid arthritis (47). Therefore, it is possible that JBT may alleviate pain by not only improving blood flow but also down-regulating inflammatory markers. This study is designed to determine whether JBT is preferable to non-pharmacologic methods for the treatment of dialysis-related myofascial pain. If this trial produces the expected results, it will provide both patients and physicians an additional option for pain control. Moreover, this trial will provide preliminary data on the effects of JBT on arm power and motor function, QOL, and safety.

HD patients may receive 10–12 medications daily, and many of these medications require multiple doses each day. Drug–drug interactions are very common due to poly-pharmacy in HD patients (48), so the involvement of JBT therapy in drug interactions is an important issue for the therapeutic efficacy and safety of medical treatment. Although several studies have reported the possibility of herb–drug interactions in CKD patients leading to nephrotoxicity, electrolyte abnormalities, or changes in kidney hemodynamics (49, 50), very few studies have tested CHM with targeted safety assessments. It is worth noting that the *Rx. Angelica Sinensis* component of JBT may increase the risk of bleeding when heparin is used to prevent clotting in the dialytic extracorporeal circuit (51). In addition, excessive intake of *Rx. Glycyrrhizae Preparata* may lead to hypertension, hypokalemia, and metabolic alkalosis (52), which may cause myopathy and arrhythmia in HD patients. Thus, the results will contribute to herb repurposing for managing myofascial pain in patients receiving HD and will provide information useful for designing a large-scale randomized controlled trial in the future.

The strengths of this study include: (1) This study is the first pilot randomized cross-over trial to evaluate the feasibility of JBT for the treatment of dialysis-related myofascial pain. (2) The design of the trial is based on TCM syndromes because dialysis-relayed myofascial pain can be inferred to inadequate *qi* and blood around the fistula arm in patients receiving hemodialysis. (3) The study of different safe and lower cost can be considered for scientific use of techniques related to TCM in dialytic patients. Nevertheless, a few study limitations should be noted. First, this study will be an open-label study because of the difficulty of preparing a suitable placebo; the pharmaceutical technology of CHM powder does not yet allow such a process because of the distinctive colors, tastes and smells of the preparations (51). Moreover, due to the use of patient-reported outcomes, the reliability of the conclusions of the study may be reduced due to the absence of participant blinding. Second, it will be a crossover trial and thus may entail possible carryover effects. The component of JBT with the longest half-life is *Rx. et Rz. Notopterygii* (*T*_1/2_ = 190–768 min); however, its washout period is only 64 h (52). To minimize this possibility, we have designed a 2-week washout period between the two groups. However, no data on the length of the washout period of JBT are currently available. The washout period involves the biological half-lives of multiple herbs and changes in the pharmacokinetics of the CHM formula (53). In the event of carry-over effects, only data from the first period will be used for the analysis, for it is a randomized parallel-group design. The current sample size was calculated on the basis of a single period of data alone; thus, the estimated size will be sufficient for specific analysis. Third, the non-treatment and JBT treatment periods will be only 4 weeks.

## Data availability statement

The original contributions presented in the study are included in the article/supplementary material, further inquiries can be directed to the corresponding author.

## Ethics statement

The studies involving human participants were reviewed and approved by Institutional Review Board at Chang Gung Memorial Hospital (approval No. 202000477A3). The patients/participants provided their written informed consent to participate in this study.

## Author contributions

M-YT and Y-YL devised the study question and design. M-YT developed the idea into the full protocol and wrote the article draft. Y-HC and H-YN reviewed the protocol. Y-CH calculated the sample size and specified the statistical strategy. M-YT and Y-TH will be in charge of enrolling participants and conducting all the procedures. All authors have read and approved the final manuscript.

## Funding

This study was funded by Chang Gung Memorial Hospital with grant number CORPG-8K0091. The funders have had no role in study design and will not have any role in the trial design, manuscript writing, or decision making for publication.

## Conflict of interest

The authors declare that the research was conducted in the absence of any commercial or financial relationships that could be construed as a potential conflict of interest.

## Publisher's note

All claims expressed in this article are solely those of the authors and do not necessarily represent those of their affiliated organizations, or those of the publisher, the editors and the reviewers. Any product that may be evaluated in this article, or claim that may be made by its manufacturer, is not guaranteed or endorsed by the publisher.

## Abbreviations

Ach: acetylcholine
AE: adverse event
AV: arteriovenous
CGMH: Chang Gung Memorial Hospital
CHM: Chinese herbal medicine
CKD: chronic kidney disease
ESRD: end stage kidney disease
FMA-UE: Fugl-Meyer Assessment for upper extremity
GMP: Good Manufacturing Practices
JBT: Juan Bi Tang
KDQOL-36: Kidney Disease Quality of Life 36-Item Short-Form Survey
MPS: myofascial pain syndrome
MTrPs: myofascial trigger points
HD: hemodialysis
QOL: quality of life
TCM: traditional Chinese medicine
VAS: visual analog scale.
